# Combination of C-Reactive Protein and Procalcitonin in Distinguishing Fungal from Bacterial Infections Early in Immunocompromised Children

**DOI:** 10.3390/antibiotics11060730

**Published:** 2022-05-29

**Authors:** Yingli Liu, Xiaoli Zhang, Tianfang Yue, Yanlai Tang, Zhiyong Ke, Yu Li, Xuequn Luo, Libin Huang

**Affiliations:** Department of Pediatrics, The First Affiliated Hospital, Sun Yat-Sen University, 58 Zhongshan 2nd Road, Yuexiu District, Guangzhou 510080, China; liuyli26@mail2.sysu.edu.cn (Y.L.); zhangxli@mail.sysu.edu.cn (X.Z.); yuetf@mail2.sysu.edu.cn (T.Y.); tangylai@mail.sysu.edu.cn (Y.T.); kezhy@mail.sysu.edu (Z.K.); liyu87@mail.sysu.edu.cn (Y.L.)

**Keywords:** invasive fungal infection, C-reactive protein, procalcitonin, children

## Abstract

Invasive fungal infection (IFI) is life-threatening in children with cancer and hematology disorders, especially when diagnosis and treatment are delayed. Conventional β-D-glucan and galactomannan tests have poor positive predictive values in the diagnosis of IFI in children with cancer. This study aims to access the diagnostic performance of C-reactive protein (CRP) and procalcitonin (PCT) in differentiating IFI from bacterial bloodstream infections in children with malignant and hematology disorders. CRP and PCT levels were measured in samples taken from patients between 12 and 24 h after fever onset, of which 24 and 102 were in the IFI and bacterial groups, respectively. We found that the CRP levels were much higher in the IFI group than the bacterial group (100.57 versus 40.04 mg/L, median, *p <* 0.001), while the PCT levels remained significantly lower (0.45 versus 1.29 μg/L, median, *p* = 0.007). Both CRP and PCT showed significant diagnostic utilities with an area under the curve (AUC) of 0.780 (95% CI, 0.664–0.896, *p* < 0.001) and 0.731 (95% CI, 0.634–0.828, *p* < 0.001) when using the cut-off values of 94.93 mg/L and 2.00 μg/L, respectively. However, the combined biomarker of CRP and PCT yielded a better diagnostic performance with an AUC of 0.934 (95% confidential interval (CI), 0.881–0.987, *p* < 0.001), which was significantly higher than that of CRP or PCT (both *p* < 0.001), with a sensitivity of 87.5% and a specificity of 87.3%. Our study demonstrates high levels of CRP combined with low PCT could differentiate IFI from bacterial bloodstream infections in immunocompromised children.

## 1. Introduction

Immunocompromised children with hematology disorders receiving chemotherapy or undergoing hematopoietic stem cell transplant (HSCT) are at high risk of infections for bacteria, fungus, virus, or other infectious agents. Invasive fungal infection (IFI) is a significant cause of morbidity and mortality. In immunocompromised children, the incidence of IFI ranges from 5.3% to 24% [1,2,3,4,5,6] and the mortality rate is approximately 18.6% to 67.6% [2,3,7,8]. Early recognition and detection are crucial in managing IFI [9,10]. However, the clinical presentation of IFI is not specific, especially in pediatric patients. The culture of blood or sterile material, as well as microscopic analysis of certain fungi are the major methods to diagnose proven IFI, but the results are mostly negative and the culture time consuming, which may delay the initiation of antifungal therapy [11,12]. In recent years, polymerase-chain-reaction (PCR)-based testing, a nucleic-acid-based diagnostic technique, has been proven to enable the possibility of early detection of IFI with high sensitivity; however, it lacks methodological standardization, and the results vary widely among laboratories [13]. Moreover, computed tomography (CT) scans are often non specific in pediatric patients with cancer who present with persistent fever, granulocytopenia, and proven pulmonary-invasive fungal disease [14].

Therefore, more attention has been paid to the biomarkers that are able to help the early diagnosis of IFI and discriminate it from bacterial infections. However, the findings are varied. The values of β-D-glucan and galactomannan assays have been proven in the diagnosis of IFI in adults, but positive predictive values are poor in children with cancer [14,15], bringing challenges for the diagnosis of IFI in children. Traditional serum biomarkers such as C-reactive protein (CRP) and procalcitonin (PCT) have also been evaluated for their abilities in distinguishing IFI and other infections. In neonates, CRP levels were shown to be significantly higher in the fungal sepsis group than in bacterial sepsis group [16], while in adult patients, they were shown not to be significantly different between the candidemia and bacteremia groups [17]. It was reported that the PCT value was markedly lower in the fungal infection group (range 0.69–1.23) than in the bacteremia group (range 4.18–12.9) at the onset of fever, and this may be a potential marker for differentiating IFI from bacterial infections, in a meta-analysis including eight studies in adult and two in neonate patients [18]. Although the pooled sensitivity and specificity estimates were 0.82 and 0.80, respectively, the ranges were very wide between the findings of the studies included. It has been doubted that PCT can effectively discriminate IFI from bacterial infections because of the low sensitivity and specificity [19]. The discordance between the findings mentioned above, including the roles of β-D-glucan, galactomannan, CRP, and PCT in IFI diagnosis, suggests that different subgroups of patients and diseases may have different responses of the biomarkers in infections.

Interestingly, two small sample studies recently showed that substantially elevated CRP combined with low PCT in immunocompromised adult patients may indicate systemic fungal infection [20,21]. However, the findings in adult patients need to be validated in immunocompromised children, including diagnostic performance and cut-off values. 

Therefore, the purpose of this study was to validate the performance of CRP and PCT in differentiating IFI from bacterial bloodstream infections, which may provide a convenient means in diagnosing IFI among immunocompromised children with malignant and hematology disorders early.

## 2. Materials and Methods

### 2.1. Study Subjects and Methods

In a retrospective study performed at our department, we analyzed clinical records of hospitalized children with hematology disorders and solid tumors ≤16 years old. The definitions of proven, probable, and possible invasive fungal infection (IFI) were according to the definitions of the European Organization for Research and Treatment of Cancer/Invasive Fungal Infections Cooperative Group and Mycoses Study Group (EORTC/MSG 2020) criteria [12]. Bacterial bloodstream infection was diagnosed in patients with fever and positive blood cultures. Children with proven or probable IFI diagnosed between December 2009 and February 2022 were analyzed, using the data of patients with bacterial bloodstream infection from January 2018 to February 2022 as a comparison. Patients suspected of having fungal and bacterial co-infections or positive cultures for contaminating pathogens without clinical evidence of infections were excluded. This study was approved by the Ethics Committee of the first Affiliated Hospital of Sun Yat-sen University in China and complied with the tenets of the Declaration of Helsinki.

The measurements CRP and PCT, as well as blood cultures were performed as part of routine testing in febrile episodes of immunocompromised children at our department. Blood samples for blood cultures were obtained at the time of fever onset or before changing antibiotics due to persistent fever. The inflammatory biomarkers including CRP and PCT were measured in fresh plasma between 12 and 24 h after fever onset. According to the laboratory interval reference of our center, elevations in CRP and PCT were defined as values greater than 10 mg/L and 0.05 μg/L, respectively.

A chest computed tomography (CT) scan was performed in children with a high suspicion of IFI or when the duration of fever was more than 3–4 days. Galactomannan antigen testing and bronchoscopy were only performed when clinically needed. 

Demographic and clinical characteristics, including age, gender, and diagnoses, were recorded. Data from initial laboratory examinations including serum PCT and CRP levels and results of the blood cultures were also collected.

### 2.2. Testing Methods

CRP was measured by an automated chemiluminescence immunoassay analyzer (Mindray M-100, Shenzhen Mindray Bio-Medical Electronics Co, Ltd., Shenzhen, China). PCT was measured on a Roche cobas e601 analyzer (Roche Diagnostics GmbH, Mannheim, Germany) using the Elecsys Prolactin II sandwich electrochemiluminescence immunoassay. Blood culture was performed by the BacT/ALERT 3D system (bioMérieux, Marcy l’Etoile, France). 

### 2.3. Statistical Analysis

Descriptive results of the continuous variables are expressed as the median interquartile ranges (IQRs). For continuous variables, differences between groups were compared using the Wilcoxon rank-sum test. Receiver operating characteristic (ROC) curves were constructed individually to determine the optimal cut-off values of CRP and PCT based on the Youden index (sensitivity + specificity − 1). The ability to diagnose IFI by the combination of biomarkers (CRP&PCT) was assessed by using automatic forward stepwise regression. The performance of CRP, PCT, and CRP&PCT was then assessed by calculating the area under the ROC curve (AUC). Comparisons of the ROC curves were performed using the DeLong et al. method. The sensitivity, specificity, positive likelihood ratio (PLR), negative likelihood ratio (NLR), positive predictive value (PPV), and negative predictive value (NPV) are reported.

All statistical analyses were performed using SPSS Version 22.0 (IBM Corp., Armonk, NY, USA) and MedCalc Version 20.100 (MedCalc Software Ltd., Ostend, Belgium). The boxplots were created with Graphpad prism Version 9.0 (GraphPad Software Inc., San Diego, CA, USA). A *p*-value <0.05 was considered significant.

## 3. Results

The baseline characteristics of the patients enrolled are shown in Table 1. Among the 126 patients, 102 (80.9%) were diagnosed with bacterial bloodstream infections and 24 (19.1%) had proven or probable IFI. *Coagulase-negative Staphylococcus* (20.6%) was the most common cause of the bacterial infections followed by *Klebsiella pneumoniae* (19.6%), *Staphylococcus aureus* 18 (17.6%), and *Escherichia coli* (15.7)*,* while *Candida spp.* were the most frequent pathogens (37.5%) in the IFI group followed by *Aspergillus* spp. (20.8%). 

**Figure 1:**
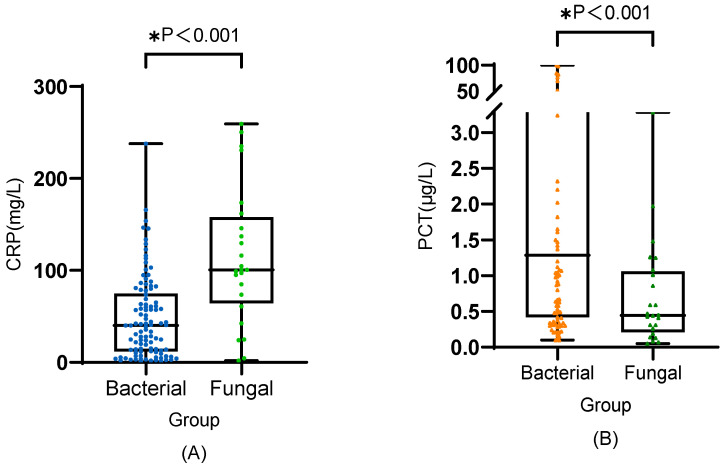
Serum CRP (**A**) and PCT (**B**) in patients with bacterial and invasive fungal infections. The differences between the bacterial group and fungal group were examined using the Wilcoxon rank-sum test. * Wilcoxon rank-sum test. CRP: C-reactive protein; PCT: procalcitonin.

As shown in Figure 1, the serum CRP level of the patients with IFI was significantly higher than that of those with bacterial infection (100.57 [64.00–157.83] versus 40.04 [11.90–74.84] mg/L, median [IQR], *p* < 0.001). The serum PCT level was much lower in the IFI group compared with that in the bacterial infection group (0.45 [0.21–1.06] versus 1.29 [0.42–13.53] μg/L, median [IQR], *p* = 0.007). 

The multiple logistic regression analysis illustrated that both CRP and PCT were independent predictors of the IFI. The details are summarized in Table 2.

**Figure 2:**
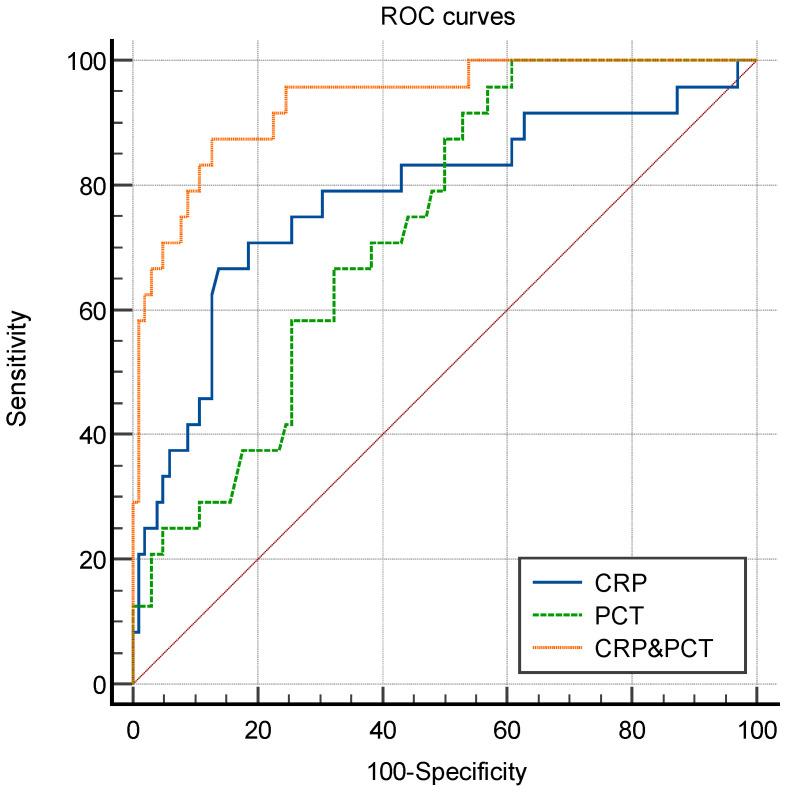
ROC curves of CRP, PCT, and CRP&PCT. ROC: receiver operating characteristic; CRP: C-reactive protein; PCT: procalcitonin.

Figure 2 depicts ROC curves for the individual biomarkers, as well as for the combination of CRP and PCT (CRP&PCT). When using the cut-off values of 94.93 mg/L and 2.00 μg/L for CRP and PCT, respectively, the AUCs for CRP, PCT, and CRP&PCT were 0.780 (95% confidential interval (CI), 0.664–0.896, *p* < 0.001), 0.731 (95% CI, 0.881–0.987, *p* < 0.001), and 0.934 (95% CI, 0.881–0.987, *p* < 0.001). The results are shown in Table 3. In addition, significant differences were found between CRP and CRP&PCT, as well as PCT and CRP&PCT (both *p* < 0.001). However, there was no statistical difference of the AUC between CRP and PCT (*p* = 0.58).

## 4. Discussion

Our study illustrates that the combination of CRP and PCT is useful in differentiating IFI from bacterial blood stream infections in febrile episodes of immunocompromised children. The sensitivity and specificity of the combination in differentiation were 87.5% and 87.3% with cut-off values of 94.93 mg/L and 2.00 μg/L for CRP and PCT, respectively. It is well known that CRP and PCT levels significantly rise after 12 and 6 h of infection, respectively. The combination of substantially elevated CRP and relatively low PCT levels tested between 12 and 24 h after fever onset strongly suggest that immunocompromised children have IFI rather than bacterial infection. This may lead to the initiation of a specific therapy in the very early stage of febrile episodes, thus reducing the mortality due to infection, including IFI. 

CRP is one of the classical acute-phase proteins, which could increase significantly at the early stage of IFI and bacterial infection [22]. In addition, the response of PCT to fungal agents is heterogeneous, and there may be a delayed increase or none in invasive aspergillosis [19]. These may explain the trend of “high CRP and low PCT” that we observed in this study in which the blood sample was obtained for testing between 12 and 24 h after fever onset.

In adult patients, β-d-glucan and galactomannan assays are accepted tools to support the diagnosis of IFI. The utility of these tests in children has been questioned. A 2017 updated guideline does not recommend using serum β-d-glucan and galactomannan in the diagnosis of IFI in children with cancer [15]. More recently, another guideline (2020) also discouraged using serum β-d-glucan in pediatric patients because of the poor predictive value of a positive test result in the available pediatric studies [14]. However, in contrast to the previous version of the guideline, the 2020 guidelines recommend galactomannan as a diagnostic tool in pediatric patients mainly based on the results of a meta analysis [23]. Although this meta analysis showed the pooled sensitivity (81% (95% CI 69–89)) and specificity (88% (95% CI 75–95)) of all 18 studies in the diagnosis of IFI, the range of both the sensitivity and specificity varied widely between the studies and from 0–100% and 35–100%, respectively. Therefore, the 2020 guidelines made additional remarks that the careful interpretation of galactomannan test results is necessary due to the test’s inherent limitations including poor positive predictive value. This highlights the importance of CRP and PCT combination in the diagnosis of IFI in pediatric patients because of the limitation of β-d-glucan, galactomannan, as well as CT scan in this population [14]. We assume that β-d-glucan and galactomannan assays combined with CRP/PCT might provide more information in the assessment of IFI in children, and further study is needed. 

There have been two small sample studies demonstrating that substantially elevated CRP combined with relatively low PCT in immunocompromised adult patients may indicate systemic fungal infection, one including 34 patients and the other 64 with hematological malignancy receiving chemotherapy or allogeneic hematopoietic stem cell trans-plantation [20,21]. Using the combination of CRP and PCT, one study showed that the sensitivity and specificity for the diagnosis of IFI were as high as 90.0% and 92.9%, respectively, for coincident PCT < 1.26 μg/L and CRP > 120.4 mg/L [21]. Our study also showed that in immunocompromised pediatric patients with a similar disease, the phenomenon of “high CRP and low PCT” can also be used to diagnosis IFI and discriminate it from bacterial infection. 

## 5. Conclusions

IFI remains an important cause of morbidity and mortality in children with cancer and hematology disorders. Early detection and diagnosis can help ensure prompt treatment and reduce mortality. In our experience, among immunocompromised children with hematologic disorders, a significant increase of CRP and low PCT help differentiate IFI from bacterial bloodstream infection, which may provide pediatric clinicians with a new option to diagnose IFI early and discriminate it from bacterial infection.

## Figures and Tables

**Table 1:** Demographic and clinical characteristics of patients.

Characteristic	Number (%)
**Age (y), median (IQR)**	5 (3, 10)
**Male**	77 (61.1)
**Primary disease:**	
Acute lymphoblastic leukemia	95 (75.3)
Acute myeloid leukemia	19 (15.1)
Mixed phenotype acute leukemia	4 (3.2)
Lymphoma	3 (2.4)
Solid tumor	4 (3.2)
Aplastic anemia	1 (0.8)
**Pathogens:**	
**Bacterial:**	102
*Coagulase-negative Staph.*	21 (20.6)
*K. pneumoniae*	20 (19.6)
*S. aureus*	18 (17.6)
*E. coli*	16 (15.7)
*P. aeruginosa*	9 (8.8)
*Streptococcus* spp.	7 (6.9)
*Enterococcus faecium*	1 (1.08)
*Gemella Berger*	1 (1.08)
*Roseomonas*	1 (1.08)
*Abiotrophia defectiva*	1 (1.08)
*Salmonella typhi*	1 (1.08)
*M.luteus*	1 (1.08)
*Citrobacter freundii*	1 (1.08)
*Propionibacterium acnes*	1 (1.08)
*Listeria monocytogenes*	1 (1.08)
*Rothia*	1 (1.08)
**Fungal:**	24
*Candida* spp.	9 (37.5)
*Aspergillus* spp.	5 (20.8)
*T.asahii*	4 (16.6)
*Mucor*	3 (12.5)
*Haematonectria haematococca*	1 (4.2)
*Trichosporon inkin*	1 (4.2)
*Pneumocystis jirovecii*	1 (4.2)

IQR: interquartile range.

**Table 2:** Multiple logistic regression analysis of biomarkers used for distinguishing IFI.

Variable	Coefficients	OR	95% CI	*p*
CRP	0.042	1.043	1.024–1.062	<0.001 ^†^
PCT	−1.029	0.357	0.140–0.914	0.032 ^†^

^†^ Multiple logistic regression. IFI: invasive fungal infection; CRP: C-reactive protein; PCT: procalcitonin; OR: odd ratio; CI: confidence interval.

**Table 3:** Performance characteristics of CRP, PCT, and the combination of CRP and PCT in diagnosing the IFI.

Biomarker	AUC	Cut-Off *	Sensitivity (%)	Specificity (%)	PLR	NLR	PPV (%)	NPV (%)
CRP	0.780 (0.664–0.896)	94.93 mg/L	66.7 (46.7–82.0)	86.3 (78.3–91.6)	4.86	0.39	53.3	91.7
PCT	0.731 (0.634–0.828)	2.00 μg/L	95.8 (79.8–99.8)	43.1 (34.0–52.8)	1.69	0.10	28.4	97.8
CRP&PCT	0.934 (0.881–0.987)	-	87.5 (69.0–95.7)	87.3 (79.4–92.4)	6.87	0.14	88.2	91.7

IFI: invasive fungal infection; CRP: C-reactive protein; PCT: procalcitonin; AUC: area under the curve; PLR: positive likelihood ratio; NLR: negative likelihood ratio; PPV: positive predictive value; NPV: negative predictive value. * The cut-off values of CRP and PCT were chosen based on the Youden index.

## Data Availability

The datasets used and/or analyzed during the current study available from the corresponding author on reasonable request.
